# Challenges and limitations in computational prediction of protein misfolding in neurodegenerative diseases

**DOI:** 10.3389/fncom.2023.1323182

**Published:** 2024-01-05

**Authors:** Marios G. Krokidis, Georgios N. Dimitrakopoulos, Aristidis G. Vrahatis, Themis P. Exarchos, Panagiotis Vlamos

**Affiliations:** Bioinformatics and Human Electrophysiology Laboratory, Department of Informatics, Ionian University, Corfu, Greece

**Keywords:** protein misfolding, structure prediction, neurodegeneration, fold recognition, deep learning algorithms

## Introduction

Proteins serve as the primary functional agents within biological systems and play an integral role in almost every aspect of cellular processes. Proteins are constructed as polymers, comprising monomers or smaller constituent units, known as amino acids. Life employs a repertoire of 20 distinct amino acids as the fundamental building blocks for the synthesis of proteins. The peptide chain possesses all the necessary covalent bonds meticulously formed within its structure (Anfinsen, 1973; Alberts et al., 2002). However, in order to fulfill its vital biological role, the peptide chain must assume a precise and distinctive conformation known as the protein's native structure. It is exclusively within this native structure that a protein can execute its designated biological functions. Proteins that deviate from their intended conformation not only fail to perform their proposed functions but may also precipitate severe consequences.

Neurodegenerative diseases frequently entail the misfolding and aggregation of particular proteins, leading to the formation of abnormal and harmful structures (Park et al., 2017). Addressing this misfolding as a therapeutic strategy presents distinct challenges in drug discovery and development (Scannevin, 2018). These challenges stem from various factors, including the dynamic nature of the protein species involved and the ambiguity surrounding which forms of a particular disease protein (such as monomers, oligomers, or insoluble aggregates) primarily contribute to cellular toxicity. Over the long term, neurodegenerative disease proteins invariably lead to synaptic dysfunction, loss, and eventual neuronal cell death (Surguchov and Surguchev, 2022). The precise mechanisms by which diverse misfolded disease proteins trigger neurotoxicity remain uncertain, and these mechanisms appear to vary depending on the particular protein species involved (Wilson et al., 2023). Misfolded disease proteins are believed to primarily exert their effects through toxic gain-of-function and/or dominant-negative effects, although instances of loss-of-function effects have also been documented. For gain-of-function mechanism a few examples could be included neurotoxic signaling, synaptic deficits, impairment of proteasomal or lysosomal degradation and axonal transport while increased vulnerability to stress, mitochondrial dysfunction and impairment of synaptic dynamic have been linked with to loss-of-function mechanism (Winklhofer et al., 2008). Proteostasis disruption is a common feature which characterizes misfolding disease proteins (Cuanalo-Contreras et al., 2013). Loss of protein functionality arises from early degradation, mislocalization or aggregation such as aggregation-prone proteins amyloid-beta, prion protein, a-synuclein and tau. Aβ oligomers are able to modulate synaptic transmission (Zhang et al., 2022), mutations in tau have been associated with microtubule destabilization and impaired axonal transport of substances (Mietelska-Porowska et al., 2014) and alpha-synuclein inhibits mitochondrial protein import machinery (Lurette et al., 2023). In Parkinson's disease the β-strand segments (β1 and β2) of α-synuclein which involved in interactions within amyloid fibrils were detected using AlphaFold2 and all-atom MD simulation (Rani et al., 2023). Focusing on human Alsin, a protein implicated in a rare neurological disorder called infantile-onset ascending hereditary spastic paralysis (IAHSP), a computational strategy based on AI-based protein structure databases was performed including structural information derived from AlphaFoldDB (Sebastiano et al., 2022). MOVA, a new in silico method for predicting variant pathogenicity using AlphaFold2 was developed and positional information for structural variants were extracted analyzing 12 ALS-causative genes (Hatano et al., 2023). Structural states of functional oligomers of all members of the KCTD family which are proteins containing a (K) Potassium Channel Tetramerization Domain with highly involvement in neurological and neurodevelopmental processes was performed (Esposito et al., 2022).

## The thermodynamic hypothesis of protein folding

A hallmark feature of a living system is the capacity for even its most intricate molecular components to self-assemble accurately and reliably. Protein folding can be broadly dissected into three interrelated facets: Folding process, mechanical aspect of folding and predicting native structures (Bryngelson et al., 1995; Dill et al., 2008). The first one encompasses inquiries into the kinetics of peptide chain folding, including the rate at which it occurs and the intermediate structural configurations that manifest between the initial conformation and the ultimate native structure. The second central to this aspect is the exploration of the underlying forces that drive and stabilize the folding process and the last one of particular relevance to biological research is the capacity to predict a protein's native structure from its constituent peptide chain.

Over the past six decades, the prevailing paradigm within the realm of protein folding has revolved around the concept that proteins undergo folding processes that result in a reduction in Gibbs free energy (expressed as a negative ΔG) (Sorokina et al., 2022). The thermodynamic hypothesis of protein folding, especially the notion that the native state represents the most stable configuration, effectively constituting the global minimum of Gibbs free energy (G), possesses an inherent allure and intuitive appeal. Moreover, this perspective significantly streamlines the development of theories and models, as it obviates the necessity to unravel the mechanisms by which a protein attains its unique native conformation. Indeed, by definition, the global minimum is a singular state. However, when the supposition is made that the native conformation occupies a local minimum rather than the global one within the Gibbs free energy landscape, it introduces a considerable degree of complexity (Lazaridis and Karplus, 2002). This complexity stems from the need to elucidate the processes by which this specific local minimum is selected from among numerous other local minima.

### Computational approaches to protein structure prediction

Computational methods for protein structure modeling are divided into distinct categories depending on the type of information they use: homology modeling and threading rely on structural data from similar proteins, whereas the ab initio method operates independently of such templates (Xu et al., 2008; Hameduh et al., 2020). Template-free approaches, followed by ab initio methods, exhibited superior performance in numerous instances. Numerous computational studies have explored solutions for protein conformation prediction, including evolutionary algorithms, Monte Carlo simulations and HP models (Istrail and Lam, 2009; Li et al., 2011; Tsay and Su, 2013). However, these approaches often struggle to efficiently search the vast conformational space of proteins.

MODELER stands out as a significant milestone among pioneering computational tools. Its primary application lies in the realm of homology or comparative modeling of protein three-dimensional (3D) structures (Webb and Sali, 2016). To harness the capabilities of MODELER, a fundamental requirement is the provision of a sequence alignment, aligning the target sequence with sequences from established, closely related protein structures referred to as templates. In return, it generates a comprehensive model that encompasses all non-hydrogen atoms within the protein structure (Kuntal et al., 2010). In addition to its fundamental function, this software offers a versatile array of supplementary features. These include the ability to perform de novo modeling of protein structure loops, assist in fold assignments, facilitate the alignment of two or more protein sequences or structures, and proficiently cluster protein structure.

**Figure 1:**
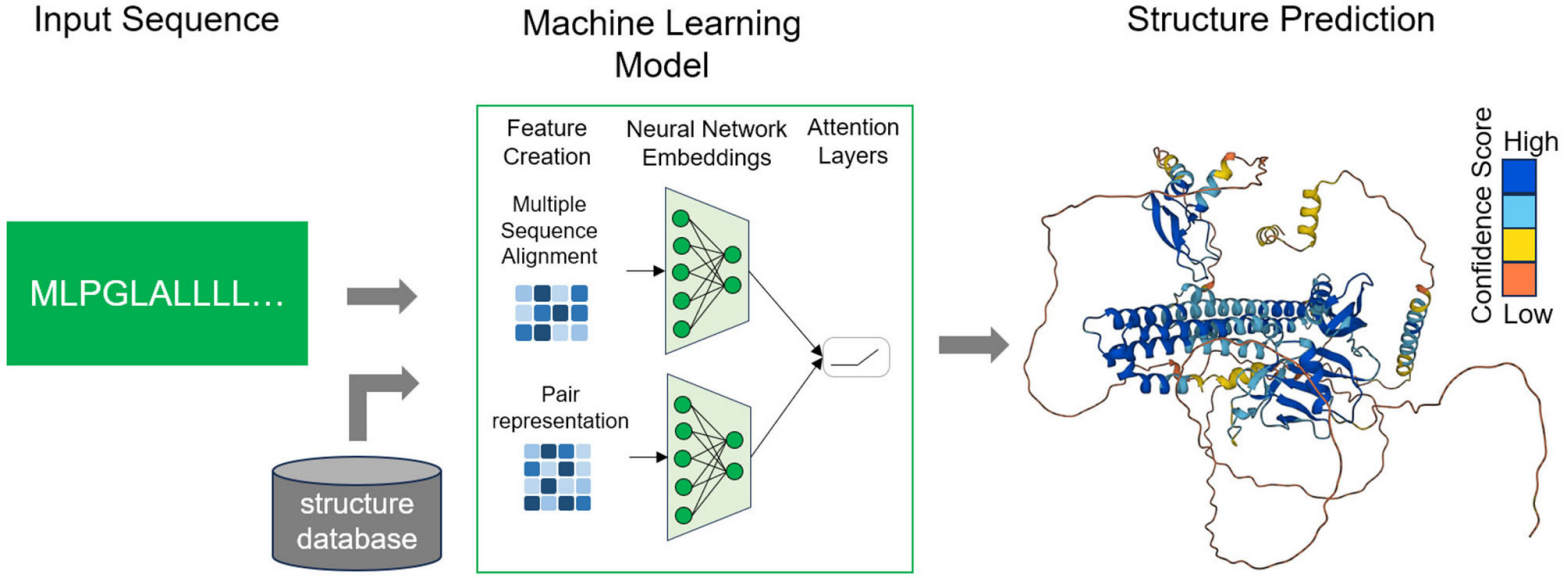
Workflow of deep learning methods. The user provides a sequence of interest and the trained model, considering in addition other structural data, provides a prediction of the folded protein structure in 3D-space. AlphaFold produces a per-residue model confidence score (pLDDT) between 0 and 100. Dark blue corresponds to very high confidence (pLDDT > 90), light blue to high (90 > pLDDT > 70), light orange to low (70 > pLDDT > 50) and dark orange to very low confidence (pLDDT < 50).

Deep learning (DL) techniques have achieved notable progress in developing end-to-end differentiable models and directly forecasting dihedral angles or 3D protein structures (Torrisi et al., 2020; Pakhrin et al., 2021) (Figure 1). However, it is important to note that these approaches, as currently reported, still heavily rely on domain-specific input features in addition to the primary amino acid sequence. This reliance on extra information can pose challenges when attempting to generalize predictions for novel protein sequences lacking any prior knowledge of these specific input features. For instance, these methods frequently require the use of templates, which are explicit sequence-to-structure mappings generated through computational procedures (AlQuraishi, 2019; Qin et al., 2020). Templates facilitate the transformation of raw amino acid sequences into three-dimensional structures by referencing existing protein structures with similar sequences. Another common reliance is on co-evolutionary data, which is built upon the assumption that pairs of residues displaying highly correlated mutations are likely to be positioned closely in the protein's three-dimensional structure (Noé et al., 2020; Xu et al., 2021).

Addressing the significant challenge posed by the enigmatic protein folding problem, meticulous organization characterizes the Critical Assessment of Protein Structure Prediction (CASP) competition. DeepMind's AlphaFold, the CASP13 winner, follows a methodology that embraces two firmly established concepts deeply ingrained in scientific literature (Senior et al., 2020). Firstly, it employs co-evolutionary analysis to map the co-variation of residues within protein sequences to physical interactions in protein structures. Secondly, it leverages deep neural networks to adeptly discern patterns in protein sequences and co-evolutionary couplings, subsequently translating them into contact maps.

In CASP14, a highly evolved iteration, denoted as AlphaFold2, emerged as the victorious solution, developed by Jumper et al. (2021). This model employs a DL network architecture that seamlessly integrates both physical and biological knowledge within a dual-track framework. To delve into the intricacies, the output of AlphaFold2 consists of the three-dimensional coordinates of all heavy molecules, complemented by a confidence score. This score is derived from an amalgamation of multiple sequence alignments (MSAs) and pairwise residue features. ColabFold was also used as an implementation of the AlphaFold framework that uses the MMseqs2 algorithm to promptly compile MSAs. Both can provide structural in specific proteins in involved in Alzheimer's disease (Santuz et al., 2022; Efraimidis et al., 2023). RoseTTAFold presents itself as an additional deep learning model that builds upon concepts initially developed by AlphaFold2. This model employs a three-track neural network, utilizing inputs at three distinct levels: the 1D sequence level, the 2D distance map level, and the 3D coordinate level, all in pursuit of protein structure prediction (Baek et al., 2021).

In another approach, the recurrent geometric network (RGN) employs a sequence of amino acids and position-specific scoring matrices (PSSMs) as its input. This method culminates in the prediction of a 3D protein structure (Chowdhury et al., 2022). Notably, the RGN model primarily relies on mathematical equations that pertain to chemical properties and employs a recurrent neural network to encode the protein sequence. Lastly, a new deep learning architecture, DeepPotential, was developed for the prediction of protein structural geometry, integrating multiple unary and pairwise features as inputs into a hierarchical deep residual neural network (Li et al., 2022).

The availability of precise protein structure prediction has driven progress across diverse fields with AlphaFold and RosettaFold exhibiting competence in the prediction of intricate protein assemblies. Similarly, several other approaches have honed their focus on the realm of protein-protein interactions (PPIs). One such method, Struct2Graph, is founded on a 3D-structure-based graph attention network, specifically tailored for PPI prediction (Baranwal et al., 2022; Soleymani et al., 2022). In essence, this technique entails the acquisition of low-dimensional feature embeddings from the graph structures of individual proteins. Another field closely related to protein folding is the effort to design novel proteins designed to possess specific desired functionalities with ProteinMPNN standing out as a noteworthy example, having demonstrated its utility in the design of monomers, cyclic oligomers, protein nanoparticles, and protein-protein interfaces (Dauparas et al., 2022).

In a parallel vein, ProteinSolver is used, underpinned by graph convolutional neural networks (CNNs), which adeptly addresses the challenge of constraint satisfaction within the realm of protein topologies (Strokach et al., 2020). This model, trained on protein sequences, acquires the capacity to discern the constraints inherent in protein structures. Subsequently, it leverages this knowledge to generate new protein sequences capable of folding into user-defined shapes.

Integrating computational predictions, such as those generated by AlphaFold or RoseTTAFold with cryoelectron microscopy (cryo-EM) data can provide a synergistic approach to achieving more accurate and reliable protein structure determination such as helicase-primase D5 (Hutin et al., 2022) and interleukin−27 signal complex (Jin et al., 2022). Emphasis should be given on inclusion of experimental information from density maps however a mechanism for assimilating this information in a form compatible with modeling should be required. In several examined cases, enhancing the deep learning prediction involves utilizing templates derived from the initial prediction and employing automatic rebuilding with the density map. This entire process can serve as an initial step in structure determination, generating a docked algorithm model that may surpass the accuracy of one predicted without the density map (Terwilliger et al., 2022).

### Limitations and improvements

AlphaFold2 gained prominence in drug discovery not just for its remarkable accuracy, but also for its contribution of predicted protein structures to a publicly accessible database (Borkakoti and Thornton, 2023). This resource has significantly simplified the drug discovery process, particularly in the context of small molecule-based research, by reducing the necessity for recurrent structure predictions. However, AlphaFold2 such as every recent DL method, lacks the capability to predict critical elements of protein structures as the algorithm is unable to directly predict three-dimensional structures solely from a raw sequence (Xu, 2019; Jumper et al., 2021). Moreover, it cannot efficiently predict intrinsically disordered proteins/regions and loops as well it showed weak performance on reverse docking (Wong et al., 2022). Although lacking stable structures, intrinsically disordered proteins (IDPs) play a vital role in numerous biological processes linked to neurodegeneration. Identification and annotation of intrinsically disordered regions (IDRs) could be precisely predicted through deep learning-based disorder predictors. Recently, DeepDPR has been proposed which can achieve satisfactory results compared with other methods in predicting IDRs consisting of four distinct steps such as Masking layer, a TimeDistributed module, a Bi-LSTM network and 4) a fully connected network (Yang et al., 2023). Current designs of deep learning models could explore a broad space of inputs and network topologies, demonstrating diversity in their approach to predicting IDRs (Zhao and Kurgan, 2022). A challenge is the combination of physicochemical attributes, considering factors such as hydrogen bonding, contact potential energy, hydrophobicity and molecular size with folding prediction through AI approaches. AlphaFold2 uses as an input amino acid sequence to generate a MSA based on several databases of protein sequences to identify which parts of the sequence are mutation prone, determining correlation between them. In this combination of bioinformatics and physical approaches, the use of physical and geometric approaches to generate features that learn from PDB data is demonstrated, leading to a network that effectively learns from the limited data available while efficiently handling the complexity and diversity inherent in structural data (Jumper et al., 2021; Bertoline et al., 2023). Similarly, RGNs require both Position Specific Scoring Matrices (PSSMs) and the raw sequence to make predictions and are currently unable to harness information pertaining to secondary protein structure (AlQuraishi, 2021). Several extensions of the AlphaFold algorithm have been created to tackle these challenges. For example, AlphaFold-Multimer trained on data with known stoichiometry, tailored for predicting multimeric interfaces while preserving a high degree of accuracy within individual protein chains while the web-based utility AlphaKnot is designed to assess entanglement within protein models resolved using AlphaFold, leveraging pLDDT confidence values (Niemyska et al., 2022; Zhu et al., 2023). Novel modeling methods derived from natural language processing for protein structure prediction have gain attention, such as ESMfold and EMBER2 to determine evolutionary, structural and functional patterns from massive protein sequence databases (Weißenow et al., 2022; Lin et al., 2023). Concurrently improving the accuracy of a predictive model could simplify the detection of required adjustments for obtaining a better match with the density map. These prospects suggest that employing an iterative approach to incorporate information from the density map into structure prediction has the potential to increase the overall modeling accuracy (Terwilliger et al., 2022). Identifying protein interactions and integrating experimental data should be taken into account to refine accuracy in predicting the structures of proteins associated with neurodegenerative diseases, such as Alzheimer's and Parkinson's disease, and better understand their underlying molecular mechanisms and their functional implications. In this direction, improvements should include enhancing the ability to predict interactions between specific proteins involved in neurodegeneration, handling intrinsically disordered proteins and proteins embedded in cell membranes, and integrating experimental data from studies like X-ray microscopy, nuclear magnetic resonance or cryo-electron microscopy. In a recent study, the ReFOLD4 refinement approach represents a significant advancement in improving AlphaFold2 models, exhibiting a commendable ability to maintain high accuracy in localized regions (Adiyaman et al., 2023).

## Conclusions

Recent progress in AI models has brought substantial changes to the study of protein folding. Predicting protein structures from sequences and exploring protein energy landscapes through simulations now heavily involve machine learning techniques. Additionally, machine learning is instrumental in designing force-fields and extracting essential information from extensive simulation datasets, enhancing the understanding of rare events like folding/unfolding transitions. While certain challenges persist, these methods are expected to play a pivotal role in advancing the field of protein folding and dynamics in the near term. Continued progress is being made in addressing more intricate unresolved questions and help exploring numerus disorders as it is widely acknowledged that protein misfolding represents a prominent hallmark in the majority of neurodegenerative diseases. Our existing knowledge base largely derives from decades of research focused on purified proteins studied in vitro, such as β-amyloid in Alzheimer's disease or a-synuclein in Parkinson's disease, and the extent of its applicability to protein folding within the intricate microenvironment of a living cell continues to be a persistent concern. Anticipating the forthcoming developments in tools and databases within the coming years, each incremental enhancement is expected to effectively address current limitations and broaden the usability and applicability of predicted protein structures.

## Author contributions

MK: Conceptualization, Writing—original draft, Writing—review & editing. GD: Conceptualization, Visualization, Writing—original draft, Writing—review & editing. AV: Visualization, Writing—original draft, Writing—review & editing. TE: Conceptualization, Supervision, Writing—original draft, Writing—review & editing. PV: Funding acquisition, Supervision, Writing—original draft, Writing—review & editing.
